# Quantitative assessment of disease markers using the naked eye: point-of-care testing with gas generation-based biosensor immunochromatographic strips

**DOI:** 10.1186/s12951-019-0493-z

**Published:** 2019-05-17

**Authors:** Qiangqiang Fu, Ze Wu, Jingxia Li, Zengfeng Wu, Hui Zhong, Quanli Yang, Qihui Liu, Zonghua Liu, Lianghe Sheng, Meng Xu, Tingting Li, Zhinan Yin, Yangzhe Wu

**Affiliations:** 1 The First Affiliated Hospital, Biomedical Translational Research Institute and School of Pharmacy and, and Guangdong Province Key Laboratory of Molecular Immunology and Antibody Engineering, Jinan University, Guangzhou, 510632 China; 2 Department of Transfusion Medicine, School of Laboratory Medicine and Biotechnology, Southern Medical University, Guangzhou, 510632 People’s Republic of China

**Keywords:** Immunochromatographic strips, Gas generation-based biosensors, C-reactive protein, Au@Pt core/shell nanoparticles

## Abstract

**Background:**

Immunochromatographic strips (ICSs) are a practical tool commonly used in point-of-care testing (POCT) applications. However, ICSs that are currently available have low sensitivity and require expensive equipment for quantitative analysis. These limitations prohibit their extensive use in areas where medical resources are scarce.

**Methods:**

We developed a novel POCT platform by integrating a gas generation biosensor with Au@Pt Core/Shell nanoparticle (Au@PtNPs)-based ICSs (G-ICSs). The resulting G-ICSs enabled the convenient and quantitative assessment of a target protein using the naked eye, without the need for auxiliary equipment or complicated computation. To assess this platform, C-reactive protein (CRP), a biomarker commonly used for the diagnosis of acute, infectious diseases was chosen as a proof-of-concept test.

**Results:**

The linear detection range (LDR) of the G-ICSs for CRP was 0.05–6.25 μg/L with a limit of detection (LOD) of 0.041 μg/L. The G-ICSs had higher sensitivity and wider LDR when compared with commonly used AuNPs and fluorescent-based ICSs. When compared with results from a chemiluminescent immunoassay, G-ICS concordance rates for CRP detection in serum samples ranged from 93.72 to 110.99%.

**Conclusions:**

These results demonstrated that G-ICSs have wide applicability in family diagnosis and community medical institutions, especially in areas with poor medical resources.

**Electronic supplementary material:**

The online version of this article (10.1186/s12951-019-0493-z) contains supplementary material, which is available to authorized users.

## Introduction

Immunochromatographic strips (ICSs) are commonly used in practical, point-of-care testing (POCT) applications, owing to their cheap cost, simplicity, portability, and ease of use [1–7]. ICSs function through the capillary action of their nitrocellulose (NC) membrane; as such, they do not require the use of additional reagents with their attendant incubation and washing steps. Currently, ICSs using gold nanoparticles (AuNPs ICSs) have been widely used in the diagnoses of a range of diseases and biological conditions, such as HIV, HBV, and early pregnancy [8–10]. However, due to their semi-quantitative, binary analysis (i.e., yes/no result) and poor detection limits (approximately 10^− 11^ M), AuNPs ICSs has limits applications in the testing of more subtle disease markers. Given this limitation, several novel ICSs have been developed that are focused on improving sensitivity and the range of detection. These new ICSs include nano-quantum dots ICSs [11], coded surface enhanced Raman scattering (SERS) nanoparticle ICSs [12, 13], electrochemical ICSs [14, 15], chemiluminescence ICSs [16], and others [17–21]. Although these ICSs have higher sensitivity and broader detection ranges, there have been restricted to use in professional laboratories and hospitals since they require expensive equipment for quantitative testing. Therefore, an ICS platform that combines high sensitivity with an easy readout is urgently needed for POCT applications.

Owing to their high sensitivity, low cost, and easy readout, gas generation biosensors have become a promising technique for POCT testing [22–27]. Currently, reported gas generation immunoassays are able to read targets through color ink movement distances. However, there are two limitations that still restrict the practical application of gas generation biosensors to POCT settings [28]. First, there is the requirement of expensive, microfluidic chip fabrication processes, and complex reagent loading as well as the addition of multiple reagents and subsequent washing steps. Second, the measured results of currently available gas generation biosensors are movement distances or pressure changes, rather than target concentrations.

Here, we have developed a novel POCT platform that integrates ICSs and gas generation biosensors (G-ICSs). In our G-ICSs, a 3D-printed reading system with a scale plate containing different concentrations of biomarkers was designed. This design allows a user to easily read a given biomarker concentration according to the position to which the color ink moved. As such, our G-ICSs greatly simplified what was once a series of complicated immunoassay steps; moreover, that routine ICSs results are offered through a gas generation-based biosensor readout. For proof of concept, we chose the C-reactive protein (CRP), which is a biomarker commonly used in the diagnosis of acute, infectious diseases. Our results showed the G-ICSs system was user-friendly; critically, it also achieved high-testing sensitivity and a visual readout. This developed G-ICSs has the potential for a wide range of rapid and quantitative POCT applications in family and community medical institutions, including clinical diagnosis, drug testing, and pathogen detection.

## Results

### G-ICSs working principle

**Fig. 1:**
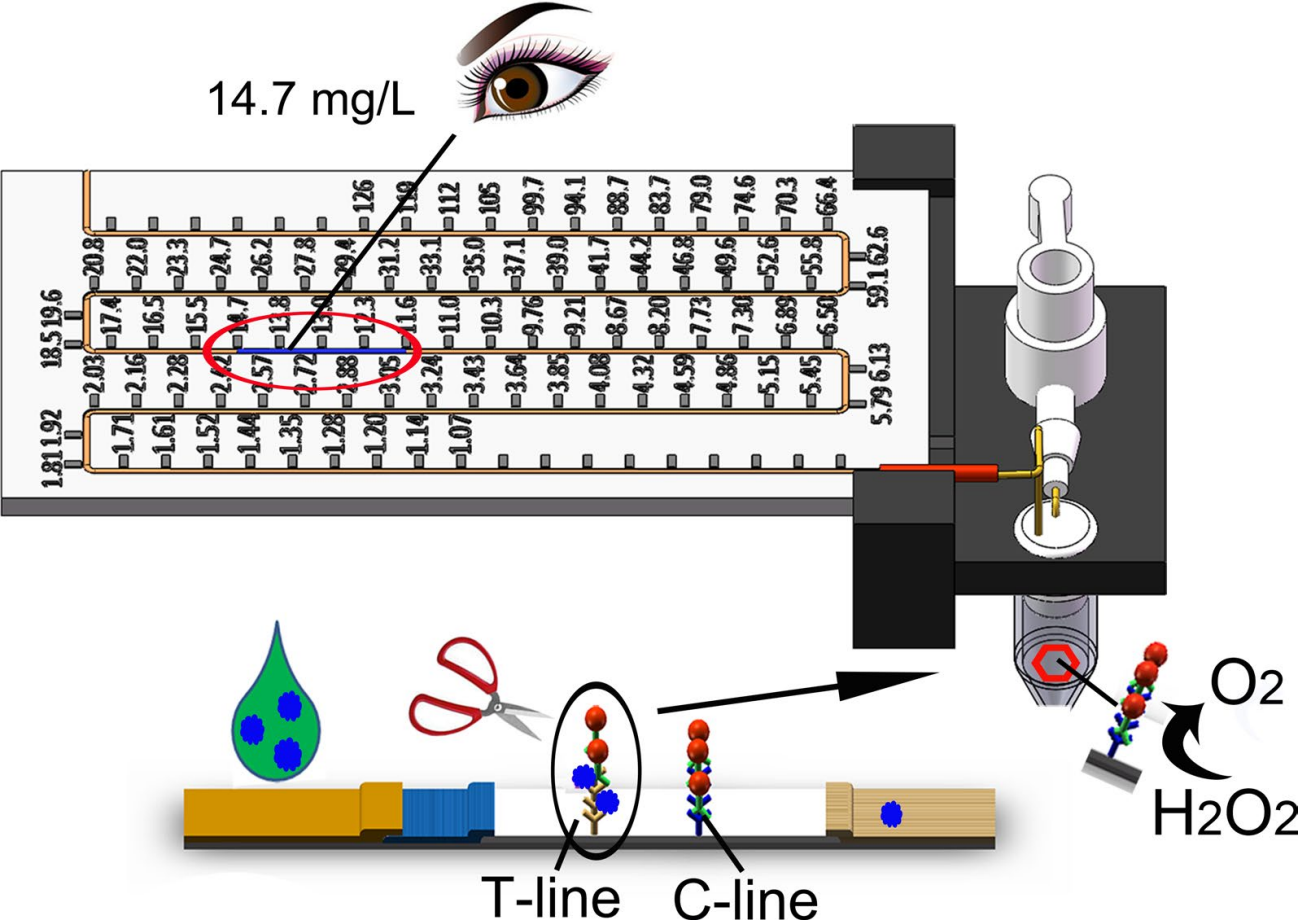
G-ICSs structure and working principle for CRP detection. Amount of mAb1-Au@PtNPs bound to the T-line increased with increasing CRP concentration. After the test, the T-lines of ICSs were separated from the G-ICSs and placed in a reaction tube containing H_2_O_2_. O_2_ generated by the mAb1-Au@PtNPs forced the blue ink forward in the polytetrafluoroethylene (PTFE) capillary. The displacement distance of the blue ink was correlated with CRP concentration. Ultimately, the user was able to directly determine CRP concentration by assessing the position of the blue ink

The G-ICSs working principle is shown in Fig. 1. The G-ICSs consisted of ICSs and the gas generation-based biosensor. A detailed assembly process for the gas generation-based biosensor is shown in Additional file 1: Figure S1. ICSs are composed of four, main parts: Sample pad for sample application (i.e., yellow-orange pad on the left side of the ICS in Fig. 1), conjugate pad for loading mAb1-Au@PtNPs (i.e., blue pad to the right of the sample pad in Fig. 1), nitrocellulose membrane for loading anti-CRP antibody 2 (mAb2; i.e., white pad in the center of the ICSs in Fig. 1), and absorbent pad serving as a liquid sink (i.e., beige pad on the far right of the ICSs in Fig. 1). These four components were then assembled on a plastic sheet.

In a routine assay, serum samples were added to the sample pad; samples then migrated to the conjugate pad. CRP within these samples bound to the mAb1-Au@PtNPs that had been pre-immobilized on the conjugate pad. Continuous flow to the nitrocellulose membrane was driven by capillary forces; CRP in samples were then captured by mAb2 in the test (T) line area. The amount of captured mAb1-Au@PtNPs by mAb2 on the T-line was positively correlated with CRP concentration. Free mAb1-Au@PtNPs moved to the calibration (C) line area, where it bound to goat anti-mouse polyclonal antibody. Excess mAb1-Au@PtNPs ultimately migrated to the absorbent pad.

To quantitatively test the amount of a given molecule of interest, the T-line areas of the G-ICSs were cut along the red line indicated on the back of the plastic mounting sheet. T-lines were then placed into a reaction tube with H_2_O_2_. Au@PtNPs catalyze the production of O_2_, gas from H_2_O_2_; the generated O_2_ forces the blue ink forward inside the polytetrafluoroethylene (PTFE) capillary. A calibration curve of G-ICSs for CRP detection was created by correlating the blue ink displacement distance with CRP concentration. Using this calibration curve, the CRP concentration corresponding to each specific blue ink distance was calculated and the concentration value was 3D-printed on the reading board. The end user could then directly read the CRP concentration using the indicated blue-ink position.

### G-ICSs optimization and characterization for CRP detection

**Fig. 2:**
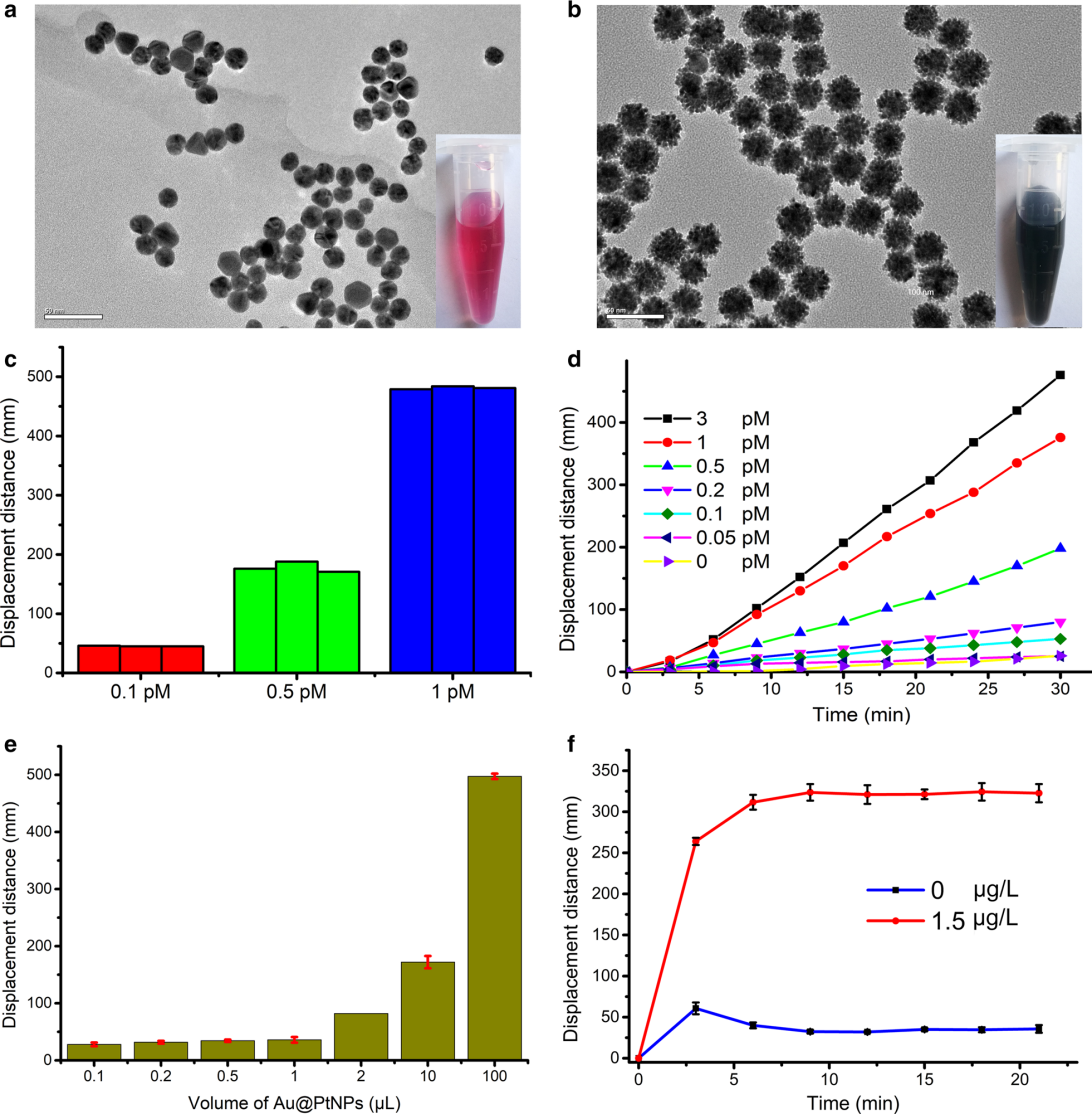
G-ICSs optimization and characterization for CRP detection.** a** Transmission electron microscopy (TEM) image of AuNPs (scale bar, 50 nm).** b** TEM images of Au@PtNPs (Scale bar, 50 nm).** c** Repeatability of gas generation-based biosensors.** d** Blue ink displacement distances as a function of O_2_ produced by different concentrations of Au@PtNPs. **e** Optimization of amount of mAb1-Au@PtNPs loaded onto ICSs conjugate pads for CRP testing. **f** Optimization of G-ICSs assay time for CRP detection. Results expressed are the means from three independent experiments

Core–shell Au@PtNPs have excellent catalytic activity and stability as well as low cost and ease of conjugation; given this, they have been extensively used in gas generation-based biosensors by reacting with H_2_O_2_ to generate O_2_ [29, 30]. Our images showed that AuNPs (diameter, 15 nm) had smooth surfaces (Fig. 2a); contrastingly, Au@PtNPs (diameter, 27 nm) had rough surfaces (Fig. 2b). These results were consistent with those found in previous studies [29], indicating the successful synthesis of Au@PtNPs. To investigate the repeatability of gas generation-based biosensors, 0.1, 0.5, and 1 pM Au@PtNPs were individually added to a solution of H_2_O_2_. Each experiment was conducted in triplicate. Standard deviations of the gas generation-based biosensors for 0.1, 0.5, and 1 pM Au@PtNPs were 3.26%, 4.62% and 1.74%, respectively. These findings demonstrated the high repeatability and stability of this approach (Fig. 2c). To validate the high catalytic activity of Au@PtNPs, different Au@PtNPs concentrations were separately added into a solution of H_2_O_2_ and the blue ink displacement distances were then recorded. Our measurements showed that even at a concentration 0.1 pM, Au@PtNPs catalyzed the time-dependent production of O_2_ from H_2_O_2_. As shown in Fig. 2d, our prepared Au@PtNPs possessed excellent catalytic activity and catalyzed O_2_ production from H_2_O_2_ in a dose- and time-dependent manner.

Since the amount of mAb-Au@PtNPs loaded onto the conjugate pad affects ICS testing sensitivity, we next sought to determine the optimal amount of mAb-Au@PtNPs to load. When loading more than 1 μL of mAb-Au@PtNPs, blue ink displacement distance increased in a dose-dependent manner. However, if less than 1 μL of mAb-Au@PtNPs were loaded, there were no noticeable changes in blue displacement distances (Fig. 2e). Therefore, we concluded that the optimal amount of mAb-Au@PtNPs to load was 1 μL. We also sought to optimize the ICS assay time (Fig. 2f). 60 μL CRP sample was dropped onto the sample pad of ICS. Over a series time, cut the T-line areas from the ICSs and placed them into 300 μL H_2_O_2_. After 30 min, the blue ink displacement distance for each assay time was recorded. When the testing time of test strip was less than 9 min, blue ink displacement distances were unstable. When the testing time of test strip was more than 9 min, blue ink displacement distances were stable. Therefore, we chose 9 min as the optimal assay time for our ICSs assessment of CRP.

### Performance of G-ICSs for CRP detection

CRP is a protein related to the nonspecific immunity found in the human immune system. In healthy adults, CRP concentration varies between 0.068 and 8.2 mg/L; however, during an infection, CRP levels increase significantly. For instance, in cases of chronic inflammation and viral infection, CRP levels can range from 10 to 40 mg/L. In cases of acute inflammation and bacterial infection, CRP levels can be even higher, ranging from 40 to 200 mg/L. Some bacterial infractions can result in even higher (200 mg/L) CRP levels [31, 32]. Therefore, CRP has been extensively used in clinical diagnoses, including for acute infectious diseases, postoperative infection monitoring, and antibiotic efficacy observation.

**Fig. 3:**
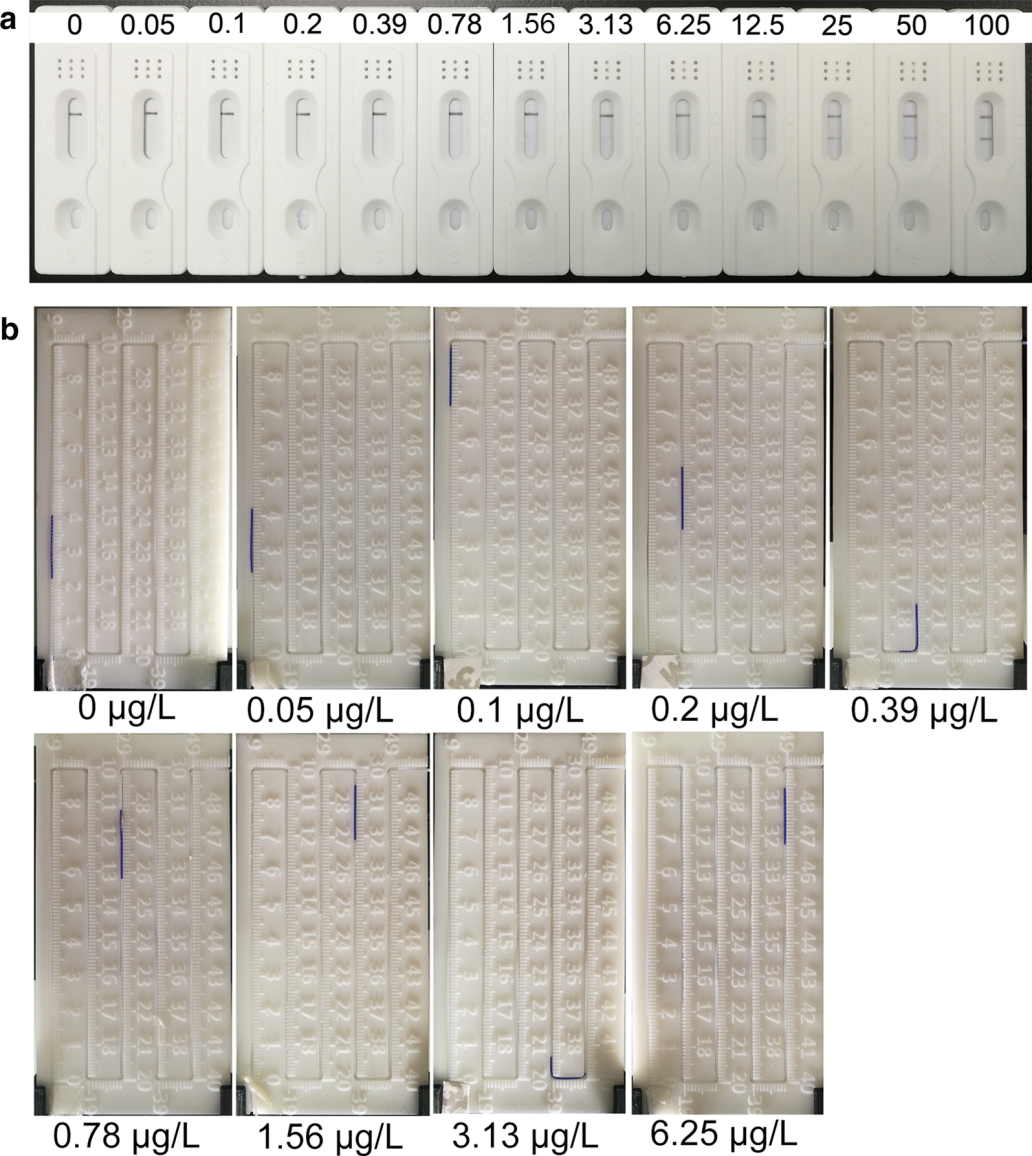
Using G-ICSs for CRP detection.** a** Images of G-ICSs used to detect different CRP concentrations.** b** CRP dose-dependent, blue ink displacement distance of G-ICSs

In this work, CRP was used as model protein to evaluate the performance of our G-ICSs biosensor. As shown in Fig. 3a, the ICSs T-line was visible only when the tested CPR concentration was greater than 6.25 μg/L. Moreover, the T-line color became darker in a dose-dependent manner. In these tests, the C-line was clearly seen in all ICSs, indicating the validity of these developed ICSs.

**Fig. 4:**
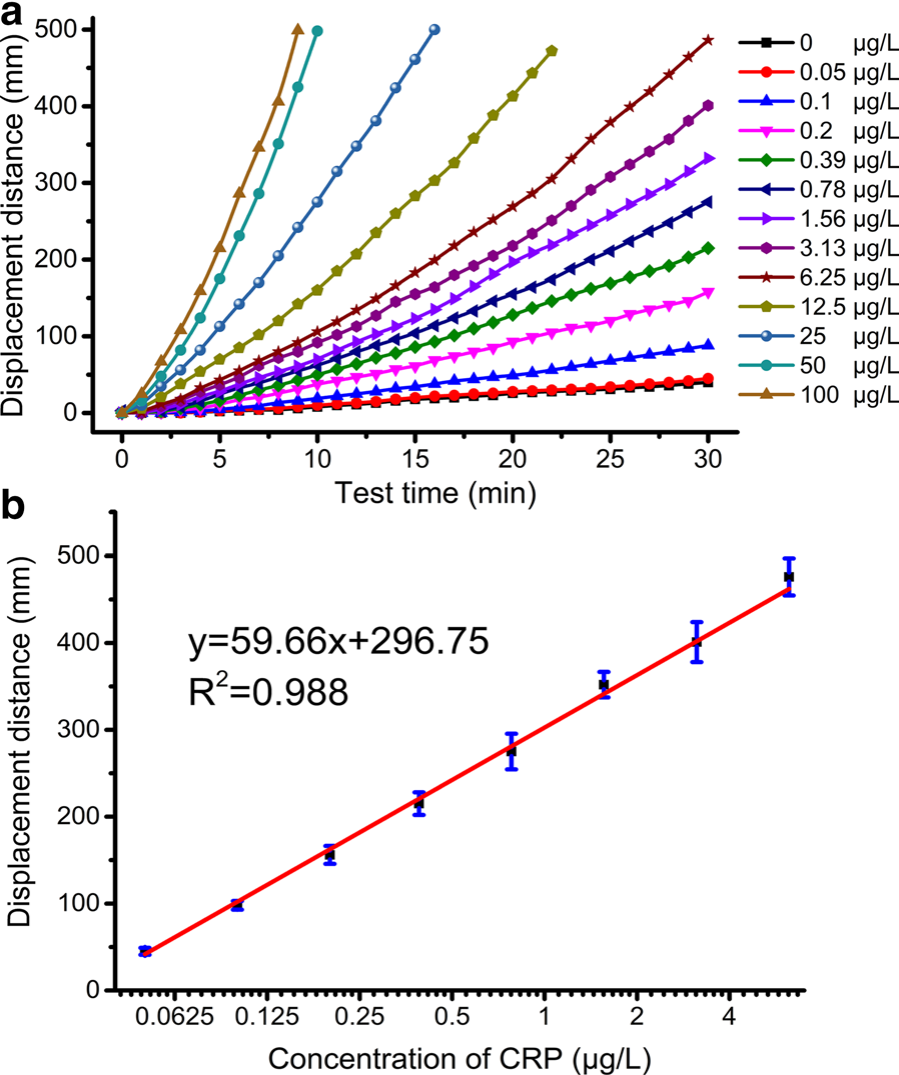
G-ICSs results from CRP testing. **a** Dose- and time-dependent displacement distance when using G-ICSs for CRP detection. **b** Standard calibration curve of G-ICSs for CRP detection. Results expressed are the means from three independent experiments

We then cut the T-line areas from the ICSs and placed them into 300 μL H_2_O_2_. After 30 min, the blue ink displacement distance for each CRP concentration was obtained. As shown in Figs. 3b and 4a, the blue ink displacement distances were dose-dependent on CRP concentration. For G-ICSs, tested CRP with concentrations greater than 6.25 μg/L generated enough O_2_ that the blue ink was pushed beyond the scale of the reading board. Collectively, our analyses showed that the CRP LDR of the G-ICSs was 0.05–6.25 μg/L; the LOD was 0.041 μg/L. Moreover, our analyses indicated that the relative standard deviation for the blue ink displacement distance for each CRP concentration was lower than 7.8%, indicating high repeatability of our developed G-ICSs for CRP detection. The developed G-ICSs were used to test 5 μg/L of CRP, bovine serum albumin (BSA), carcinoembryonic antigen (CEA), ovalbumin (OVA) and hemoglobin. Result showed blue ink movement distances of G-ICSs for 5 μg/L of CRP was 185 mm, whereas blue ink movement distances of G-ICSs for 5 μg/L of BSA, CEA, OVA and hemoglobin were lower than 55 mm (Additional file 1: Figure S8). These results indicating platform specifically capture CRP rather than binding any other targets in solution.

### G-ICS storage stability

We next sought to investigate G-ICS storage stability. G-ICSs were stored at 37 °C; every seven days, two G-ICSs were randomly selected and used to detect CRP (0 and 5 μg/L samples). Results showed that there was no degradation in the ability of the stored G-ICSs to cause blue ink displacement (Additional file 1: Figure S7). These results clearly demonstrated the high-storage stability of G-ICSs.

### Comparison of G-ICSs CRP detection with fluorescent- and gold AuNP ICSs

We next compared the sensitivity of three types of ICSs: G-ICSs, fluorescent ICSs, and AuNPs ICSs. This was done by using each to detect CRP using similar methodologies and antibodies. Results indicated that the fluorescent ICS LDR was 0.78–50 μg/L with an LOD of 0.58 μg/L (Additional file 1: Figures S3, S4). The AuNPs ICS LDR was 3.13–50 μg/L with an LOD of 2.53 μg/L (Additional file 1: Figures S5, S6). Given these results, we concluded that our developed G-ICSs had both a higher sensitivity and wider detection range when compared with either fluorescent or AuNPs ICSs. Although fluorescent and AuNPs ICSs both meet the sensitivity threshold needed for CRP detection, our developed G-ICSs provide a more sensitive range for the detection of trace amounts of CRP. In other words, our G-ICSs can detect levels of disease that fluorescent and AuNPs ICSs are unable to.

### Using G-ICSs to detect CRP levels in serum

**Fig. 5:**
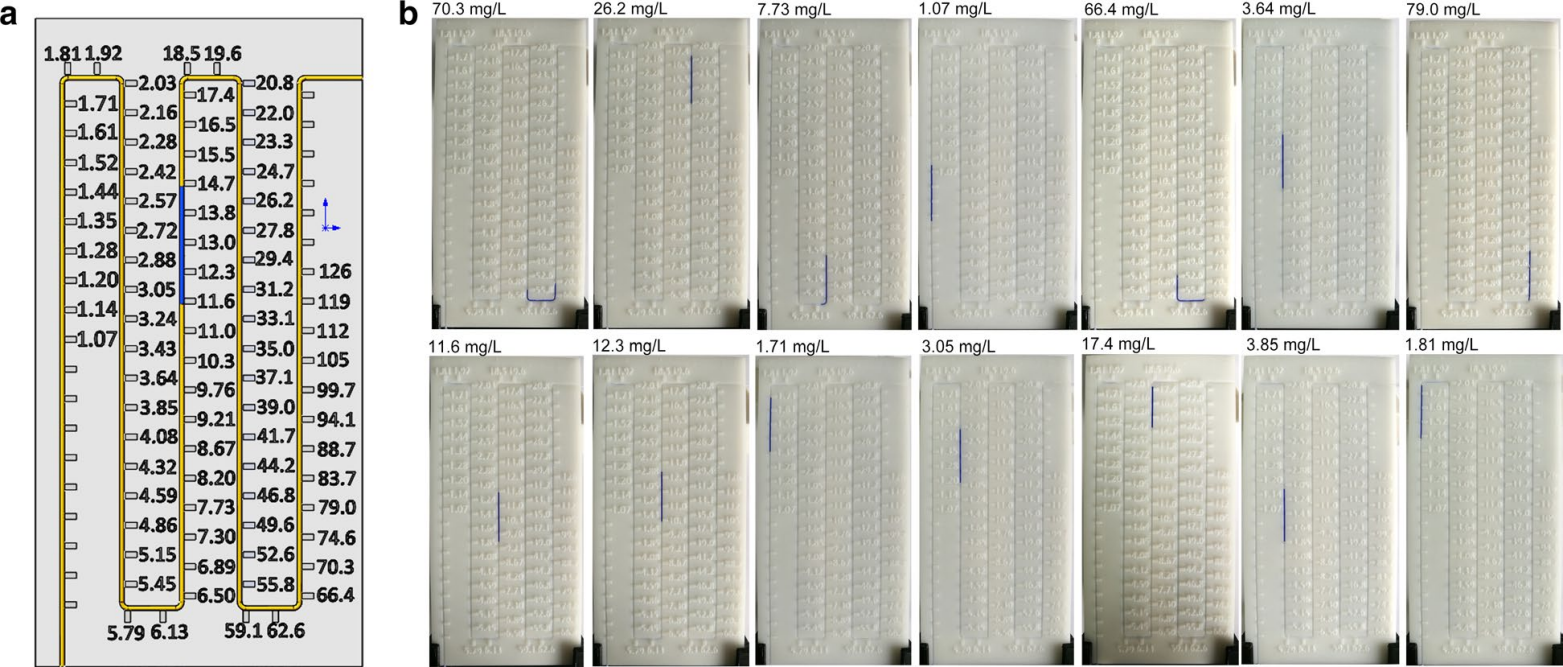
Using G-ICSs to test CRP in serum samples.** a** 3D model of the reading board. The unit of scale is mg/L.** b** Results after using G-ICSs to test for CRP in serum samples. Values above each image are CRP concentrations in serum samples as detected by a chemiluminescent (CL) immunoassay

We sought to confirm the practical usability of G-ICSs by assaying the CRP concentrations from 14 serum samples. Using our G-ICSs, CRP concentrations were directly read according to the blue ink position on the reading board (Fig. 5). Critically, our G-ICSs eliminate the need for auxiliary equipment or complicated computations. To match the linear detection range of the CRP G-ICSs, serum samples were pre-diluted 20,000 times; the values of the actual CRP concentration required multiplying the reading board value by 20,000. Errors from the reading board were less than 3.07%, as calculated according by (Vn − Vn − 1)/(2 * Vn) * 100%, where v_n_ is the value of scale n and v_n−1_ is the value of scale n − 1. Serum CRP levels were also tested using a chemiluminescent (CL) immunoassay. Results showed that the coincidence rates between the G-ICSs and CL immunoassay ranged from 93.72 to 110.99% (Table 1). Finally, serum samples with very low concentrations CRP (0.67 mg/L, 0. 72 mg/L, 0.81 mg/L) were tested by G-ICSs. Blue ink movement distances of G-ICSs for these serum samples testing were no significant difference compared with control group (0 mg/mL CRP in PBS buffer) (Additional file 1: Figure S9). This results demonstrating non-specific binding occurs when using G-ICSs to detect CRP at serum. These results indicated that our G-ICSs were reliable and repeatable and have potential use in CRP POCT testing.

**Table 1:** Comparison of CRP test in serum samples by G-ICSs and CL immunoassay

Sample number	1	2	3	4	5	6	7	8	9	10	11	12	13	14
CL immunoassay	84.57	25.43	6.76	1.21	59.99	3.43	64.17	10.25	11.64	1.78	3.14	15.83	3.59	1.91
G-ICSs														
Mean (mg/L)	80.8	23.80	7.50	1.16	58.10	3.50	69.33	11.40	11.83	1.78	3.24	15.60	3.71	1.88
S.D. (mg/L)	11.53	2.15	0.45	0.08	5.07	0.12	8.58	0.35	0.40	0.06	0.34	1.56	0.12	0.06
CV (100%)	14.27	9.02	6.07	6.49	8.73	3.46	12.38	3.04	3.41	3.25	10.49	9.99	3.27	3.37
Recovery (100%)	95.5	93.72	110.99	95.59	96.84	102.04	108.05	108.37	101.66	99.81	103.40	98.55	103.34	98.60

Mean: Average of CRP concentrations in serum samples test by the G-ICSs (n = 3). S.D.: Standard deviation of CRP concentration in serum samples test by the G-ICSs (n = 3). Coefficient of variation (CV) = (S.D./Mean) × 100%. Recovery (%) = (Results of G-ICSs/Results of CL)*100%

## Conclusions

We present here a novel POCT platform that integrates a gas generation biosensor with an Au@PtNPsbased ICSs (G-ICSs). With this G-ICSs, complex immunoassay procedures were greatly simplified and allowed for the easy, naked-eye reading of target molecule concentrations without the need for auxiliary equipment, instruments, or complicated computations. CRP concentrations from 14 serum samples were tested using our developed G-ICSs. Our results showed that the coincidence rate between G-ICSs and a CL immunoassay ranged from 93.72 to 110.99%. These results demonstrated that our G-ICSs were reliable and repeatable in real, POCT applications. G-ICSs have potential applications in clinical diagnoses (e.g., community medical institutes), personalized medicine, and environment and food safety monitoring.

## Materials and methods

### Reagents and materials

Streptavidin-conjugated anti-CRP monoclonal antibodies, goat anti-mouse polyclonal antibody, and CRP protein were all obtained from Shanghai Lingchao Biotech (Shanghai, China). Chloroauric acid (HAuCl_4_·3H_2_O) and Chloroplatinic acid (H_2_PtCl_6_·6H_2_O) were bought from Amresco (USA). Methoxypolyethylene glycol thiol (Biotin-mPEG-SH) was bought from JenKem Technology Co., Ltd. (Beijing, China). Bovine serum albumin (BSA) was purchased from Shanghai Seebio Biotech, Inc (Shanghai, China). Tween-20 was purchased from Sigma (St. Louis, MO, USA). Sodium chloride, potassium chloride, disodium hydrogen phosphate, and potassium dihydrogen phosphate were all obtained from SINOPHARM (Shanghai, China). Hydrogen peroxide (H_2_O_2_, 30 wt%), Coomassie brilliant blue (CBB) G-250, and all other reagents were purchased from GZ Chemical Reagent (Guangzhou, China). Steel microtubules (Ø 0.4 mm × 0.15 mm), polytetrafluoroethylene (PTFE) capillaries (Ø 0.3 mm), silica gel plugs (Ø 7 mm), and miniature valves (Ø 2 mm) were purchased from Pureshi (Shanghai, China). All aqueous solutions were prepared using Milli-Q water.

### Equipment

A Photocurable 3D printer was purchased from the Shenzhen CREALITY 3D. Ltd (DP002) (Shenzhen, China) and a fused deposition modeling (FDM) 3D printer was purchased from the SHINING 3D (e-star 3) (Hangzhou, China). Other equipment included a field-emission transmission electron microscope (TEM, Philips, Holland), centrifuge (Beckman, Germany), XYZ 3200 series dispense system (Bio-Dot Scientific Equipment, Pvt. Ltd.), and a programmable HGS201 strip cutter.

### Au@PtNPs preparation

The Au@PtNPs were synthesized according to our previously reported method [29]. In brief, the preparation of AuNPs used 4 mL of 1% HAuCl_4_ solution and 96 mL ultrapure water. These components were mixed in a round-bottom flask with a reflux condenser; 3 mL of 4% sodium citrate was then added into the boiled solution, followed by boiling and stirring for 20 min. The resulting AuNPs were a wine-red color.

To prepare the Au@PtNPs, we did the following: 10 mL AuNPs and 200 μL of 3.86 mM H_2_PtCl_6_ were mixed and heated to 80 °C with magnetic stirring. 800 μL aliquot of 10 mM ascorbic acid was slowly dropped into this mixture over the course of 3 min. T the mixture was then heated and stirred for another 30 min.

### mAb1-Pt@AuNPs preparation

mAb1-Au@PtNPs were prepared according to our previously published work [23]. Briefly, 10 μL of 1% (wt%) Tween 20 and 5 μL of 100 μM biotin-mPEG-SH were added to 1 mL Au@PtNPs. 10 μL of 120 μM biotin-mPEG-SH linker was then added, followed by the addition of 50 μL 0.2 M H_3_PO_4_. After 1 h of incubation at 37 °C, Au@PtNPs were centrifuge-washed three times at 13,000 rpm, then resuspended in 1 mL PBST buffer (0.1 M phosphate buffered saline, pH 7.4, containing 0.05% Tween 20). 5 μL of 1 mg/mL mAb1-streptavidin was then added to the Au@PtNPs and incubated for 30 min. The resulting mAb1-Au@PtNPs were centrifuge-washed at 13,000 rpm for 15 min, then resuspended in 1 mL phosphate buffered saline (PBS) buffer (0.1 M phosphate buffered saline, pH 7.4) for storage.

### ICSs preparation for CRP test

Coating of mAb2 onto the nitrocellulose membrane was performed as follows: Anti-CRP-antibody (1 mg/mL) was coated on the specific area of the nitrocellulose membrane as the T-line using an automatic dispenser. 1 μL of mAb2 was dispensed onto 1 cm of the nitrocellulose membrane. To conveniently cut the T-line after the experiment, a red line was drawn on the back of the plastic mounting sheet along the T-line using programmable XYZ 3200 series dispense system. The coated nitrocellulose membrane was then immediately placed in an oven at 37 °C for a minimum of 24 h for later use.

Treatment of the sample pad was performed as follows: 5 mL PBS buffer containing 25 mg BSA, 50 μL Tween-20, and 100 mg polyethylene glycol 4000 (PEG4000) was dropped on a 30 cm length sample pad. The sample pad was then oven-dried at 37 °C. mAb1-Pt@AuNPs was dispensed onto the conjugate pad using an automatic dispenser. Sample pads, conjugate points, nitrocellulose membrane, and the absorbent pad were all mounted onto the plastic adhesive plate in sequence with 2 mm overlaps. The resulting construct was cut into 4 mm ICSs and placed into plastic housings.

### Assembly of gas generation-based biosensor device

Components of the gas generation-based biosensor included a 3D-printed holder, reading board, PTFE capillary, air valve, silicon tube, fine-iron pipe, and centrifuge tube. All components were assembled as shown in Fig. 1. Detailed, step-by-step assembly instructions are provided in Additional file 1.

For each test, we drew 20 mm blue ink (10 mg Coomassie brilliant blue dissolved in 1 mL ethanol) into a 600 mm PTFE capillary. This was then inserted into the silicone tube, with the inside end of the blue ink aligned at the starting scale. The PTFE capillary was embedded in the groove of the reading board. After each test, the capillary was replaced. For the CRP test, a standard calibration curve was first created. Scales of the reading board corresponded to the displacement distance (in millimeters) of blue ink. If the serum samples required dilution prior to testing, the final CRP concentration was equal to the value on the reading board multiplied by the dilution ratio.

### G-ICSs calibration using pure CRP samples

60 μL of different concentrations of CRP samples were dropped onto the sample pads of the ICSs. After 12 min, the T-line area on the nitrocellulose membrane was cut along the red line on the back of the plastic sheet and placed into a reaction tube containing 300 μL of H_2_O_2_ (30%). The reaction tube was immediately connected to the silicone plug of the readout device and the air valve was closed. The blue ink displacement distance in the PTFE capillary was then recorded. The calibration curve of the G-ICSs for CRP was established by correlating the CRP concentration with blue ink displacement distance.

### Visual quantification of CRP in serum samples using G-ICSs

Serum samples were diluted 20,000 times using PBS; 60 μL of diluted serum samples were then dropped on the sample pad of the ICSs. After 12 min, the T-line area of the ICSs was cut and put into a reaction tube containing 300 μL H_2_O_2_. The reaction tube was then connected to the silicone plug of the gas generation-based biosensors and then air valve was closed. After 30 min, CRP concentration was recorded according to the position of blue ink on the reading board.

### Chemiluminescent (CL) immunoassay for CRP testing

This section was displayed in Additional file 1.

### Preparation of AuNPs ICSs and fluorescence ICSs for detection of CRP

A detailed method and procedures regarding the preparation of fluorescent and AuNPs ICSs are found in Additional file 1.

## Additional file

**Additional file 1: Figure S1.** Assembly process of gas generation based biosensors. **Figure S2.** TEM results of AuNPs. **Figure S3.** Concentration-dependant fluorescence intensities of ICSs for CRP test. **Figure S4.** Calibration curve of fluorescence ICSs for CRP test. **Figure S5.** Results of AuNPs ICSs for CRP test. **Figure S6.** Calibration curve of AuNPs ICSs for CRP test. **Figure S7.** Storage time of G-ICSs for CRP test. **Table S1.** Comparison of CRP test in serum samples by G-ICSs and chemiluminescent immunoassay (CL). **Figure S8.** Specificity of G-ICSs for CRP testing. **Figure S9.** Non-specific binding occurs when using G-ICSs to detect CRP at serum.

## Data Availability

All data generated and analyzed during this study are included in this published article.
