# Supplementary material for: Quantitative assessment of disease markers using the naked eye: point-of-care testing with gas generation-based biosensor immunochromatographic strips
Source: J Nanobiotechnology. 2019 May 17;17:67. doi: 10.1186/s12951-019-0493-z (PMC6524263; doi:10.1186/s12951-019-0493-z)
Supplement: Supplementary file 1 — Additional file 1: Figure S1. Assembly process of gas generation based biosensors. Figure S2. TEM results of AuNPs. Figure S3. Concentration-dependant fluorescence intensities of ICSs for CRP test. Figure S4. Calibration curve of fluorescence ICSs for CRP test. Figure S5. Results of AuNPs ICSs for CRP test. Figure S6. Calibration curve of AuNPs ICSs for CRP test. Figure S7. Storage time of G-ICSs for CRP test. Table S1. Comparison of CRP test in serum samples by G-ICSs and chemiluminescent immunoassay (CL). Figure S8. Specificity of G-ICSs for CRP testing. Figure S9. Non-specific binding occurs when using G-ICSs to detect CRP at serum. [file 12951_2019_493_MOESM1_ESM.docx]

**Additional material**

#### Naked Eyes Quantitative Test Disease Markers by Gas Generation biosensors with Au@Pt Core/Shell Nanoparticle based Immunochromatographic Strips

Qiangqiang Fu^a^, Ze Wu^b^, Jingxia Li^a^, Zengfeng Wu^a^, Hui Zhong^a^, Quanli Yang^a^, Qihui Liu^a^, Zonghua Liu^a^, Lianghe Sheng^a^, Meng Xu^a^, Tingting Li^b, *^, Zhinan Yin^a, *^, Yangzhe Wu^a, *^

^a^ The First Affiliated Hospital, Biomedical Translational Research Institute and Guangdong Province Key Laboratory of Molecular Immunology and Antibody Engineering, Jinan University, Guangzhou, 510632 Guangdong, P.R. China.

^b^ Department of Transfusion Medicine, School of Laboratory Medicine and Biotechnology, Southern Medical University, Guangzhou 510632, PR China.

^*^Corresponding authors: [apple-ting-007@163.com](mailto:apple-ting-007@163.com); [zhinan.yin@yale.edu](mailto:zhinan.yin@yale.edu); [tyzwu@jnu.edu.cn](mailto:tyzwu@jnu.edu.cn)

**Reagents and materials**

Anti-CRP monoclonal antibodies (mAb1 was conjugated with streptavidin) and CRP protein were obtained from Shanghai Lingchao Biotech (Shanghai, China). Methoxypolyethylene glycol thiol (mPEG-SH) was obtained from JenKem Technology Co., Ltd. (Beijing, China). Bovine serum albumin (BSA) was purchased from Shanghai Seebio Biotech, Inc (Shanghai, China). Tween-20 and PVP was purchased from Sigma (St. Louis, USA). Chloroauric acid (HAuCl_4_·3 h_2_O) were obtained from Amresco (USA). Carboxyl fluorescent microspheres (FMs, the excitation light is 365nm and the emission light is 605 nm) were purchased from Shanghai Huizhi Biotech (Shanghai, China). Nitrocellulose (NC) membranes (HFB13504) were purchased from Millipore (Shanghai, China). PVC sheets, conjugation pads, absorbance pads were purchased from local market (Shanghai, China). Potassium chloride, disodium hydrogen phosphate, potassium dihydrogen phosphate were all obtained from SINOPHARM (Shanghai, China). Other commonly used reagents were purchased from GZ Chemical Reagent (Guangzhou, China). All aqueous solutions were prepared using Milli-Q water.

**Preparation of fluorescence ICSs for CRP test**

**Conjugation of FMs with mAb1**: 100 μL FMs was dispersed in 400 μL of distilled water. After 60 μL, 1 mg/mL EDC and 40 μL 1] mg/mL NHS were added, the solutions were incubated at room temperature for 30 min. The resulting solutions were centrifuged at 10000 rpm for 15 min at 4 °C and then the supernatant was discarded, and the process was repeated three times. The resulting pellet was suspended in 500 μL (pH 7.2) PBS and then mixed with 100 μL 0.1 mg/mL mAb1. The resulting solution was incubated at room temperature for 2 h and then 100 μL 100 mg/mL BSA was used to block excess carboxyls of FMs for 1 h. The resulting solutions were centrifuged at10000 rpm for 15 min at 4 °C and the supernatant was discarded to eliminate the unbounded BSA. The FMs-mAb1 was suspended in 200 mL 0.01 M pH 7.2 PBS buffer and then stored at 4 °C.

**Assembly of ICSs for CRP test**: mAb2 (1 mg/mL) was dispensed on the specific area of the NC membrane as the test line (T-line) by an automatic dispenser. The volume was 1 μL mAb2 dispensed on 1 cm NC membrane. The mAb2 coated NC membrane was dried at 37 °C at least 24 h. Treatment of sample pad: 5 mL treatment buffer (PBS (0.15 M, pH 7.4) containing 0.5% BSA), and following dried at 37 °C. FMs-mAb1 was dispensed on the conjugate pad using the automatic dispenser with a volume of 1.5 μL/cm. Sample pads, conjugate points, NC membrane and absorbent pad are pasted on plastic adhesive plate in sequence with 2 mm overlaps. This stick was cut into 4 mm ICSs and following placed into plastic housings.

**Test CRP use the fluorescence ICSs**: 60 μL CRP samples were added to samples pad of fluorescence ICSs. After 15 min, Results of fluorescence ICSs was recorded by the fluorescence ICSs reader.

**Preparation of AuNPs ICSs for CRP test**

**Conjugation of AuNPs with mAb1:** Using 0.25 M K2CO3, the pH of AuNPs was adjusted to 8.5. Then 5 μL of mAb1 (1.0 mg/mL) was added drop by drop to 1 mL of pH adjusted AuNPs. This mixture then gently stirred for 30 min, followed by blocking with 0.5 mL of 1% BSA solution for 30 min and centrifuging at 10,000 rpm for 15 min. After centrifugation, the pellets were suspended in 0.25 mL of dilution buffer [20mM Tris/HCl buffer (pH 8.2) containing 1% (w/v) BSA], and then stored at 4 °C.

**Assembly of ICSs for CRP test**: mAb2 (1 mg/mL) was dispensed on the specific area of the NC membrane as the test line (T-line) by an automatic dispenser. The volume was 1 μL mAb2 dispensed on 1 cm NC membrane. The mAb2 coated NC membrane was dried at 37 °C at least 24 h. Treatment of sample pad: 5 mL treatment buffer (PBS (0.15 M, pH 7.4) containing 0.5% BSA), and following dried at 37 °C. AuNPs-mAb1 was dispensed on the conjugate pad using the automatic dispenser with a volume of 1.5 μL/cm. Sample pads, conjugate points, NC membrane and absorbent pad are pasted on plastic adhesive plate in sequence with 2 mm overlaps. This stick was cut into 4 mm ICSs and following placed into plastic housings.

**Detection of CRP use the AuNPs ICSs**: 60 μL CRP samples were added to samples pad of AuNPs ICSs. After 15 min, Results of fluorescence ICSs was recorded.

**Chemiluminescent (CL) immunoassay for CRP testing**

The protocol used for the chemiluminescent (CL) immunoassay to assess CRP levels was as follows: Each well of the microplate was coated with 1 μg of anti-CRP-mAb1 and incubated 3 h at 37℃. Unbound mAb1 was removed from the plate using three washes with PBST buffer. Each well was then blocked with 5% BSA in PBST at 37℃ for 1 h. 100 μL CRP standard (0 pg/mL, 10 pg/mL, 20 pg/mL, 40 pg/mL, 80 pg/mL, 160 pg/mL, 320 pg/mL) were separately added to each well and incubated for 30 min at 37℃. After removing the unbound CRP with another series of PBST washes, 100 μL of HRP-labelled mAb2 conjugate was added and incubated at 37℃ for 30 min. The microplate was then washed three times using PBST to remove unbound the HRP-mAb2. 100 μL of chemiluminescent substrate was added into the microwell and CL intensity was measured using a multimode reader. The calibration curve for the CRP CL immunoassay was established according to the relationship between CRP concentration and CL intensity.

For detection of CRP in serum samples, 20,000-time diluted serum sample were added to each well and incubated for 30 min at 37℃. After removing the unbound CRP with PBS washes, 100 μL of HRP-labelled mAb2 conjugate was added and incubated at 37℃ for 30 min. The microplate was then washed three times using PBST to remove any unbound HRP-mAb2. 100 μL of CL substrate was then added into the microwell and CL intensity was measured using a multimode reader. The CRP concentration was calculated according to the measured CL intensity and a calibration curve established using these results.

**
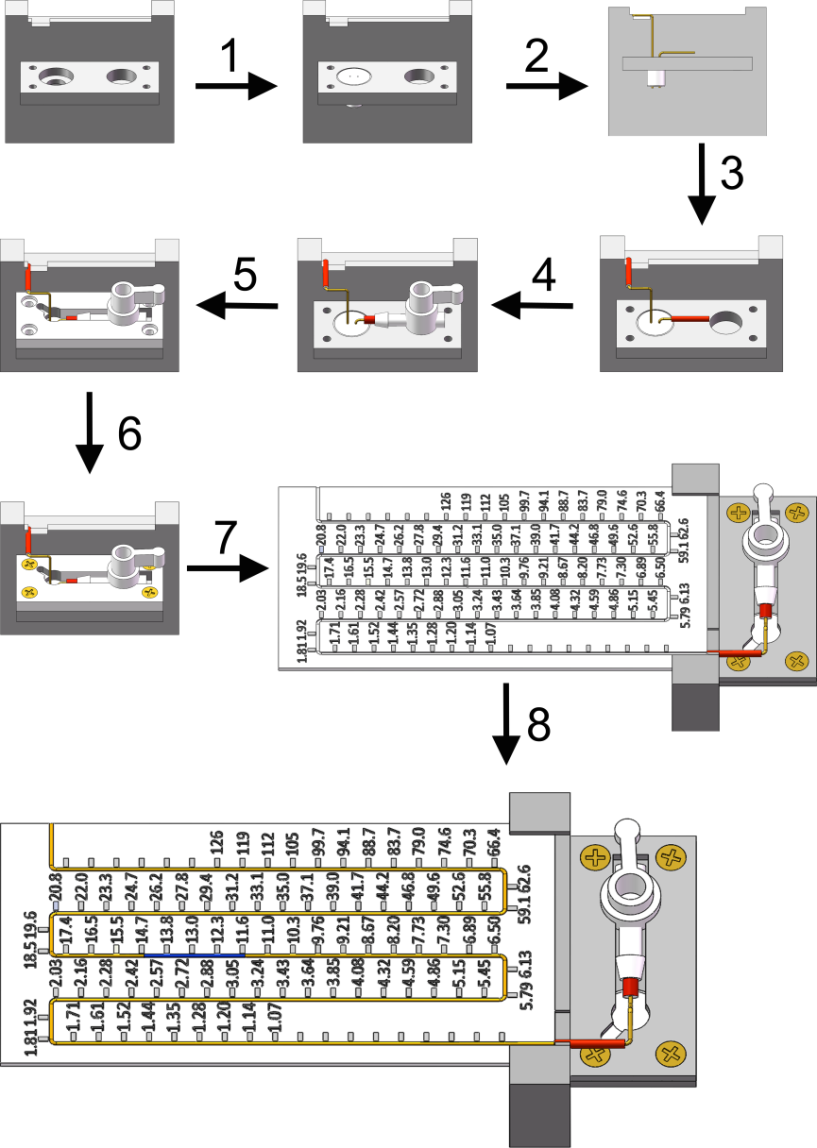
Results**

**Figure S1**. Assembly process of gas generation based biosensors.





**Figure S2**. TEM results of AuNPs.

**
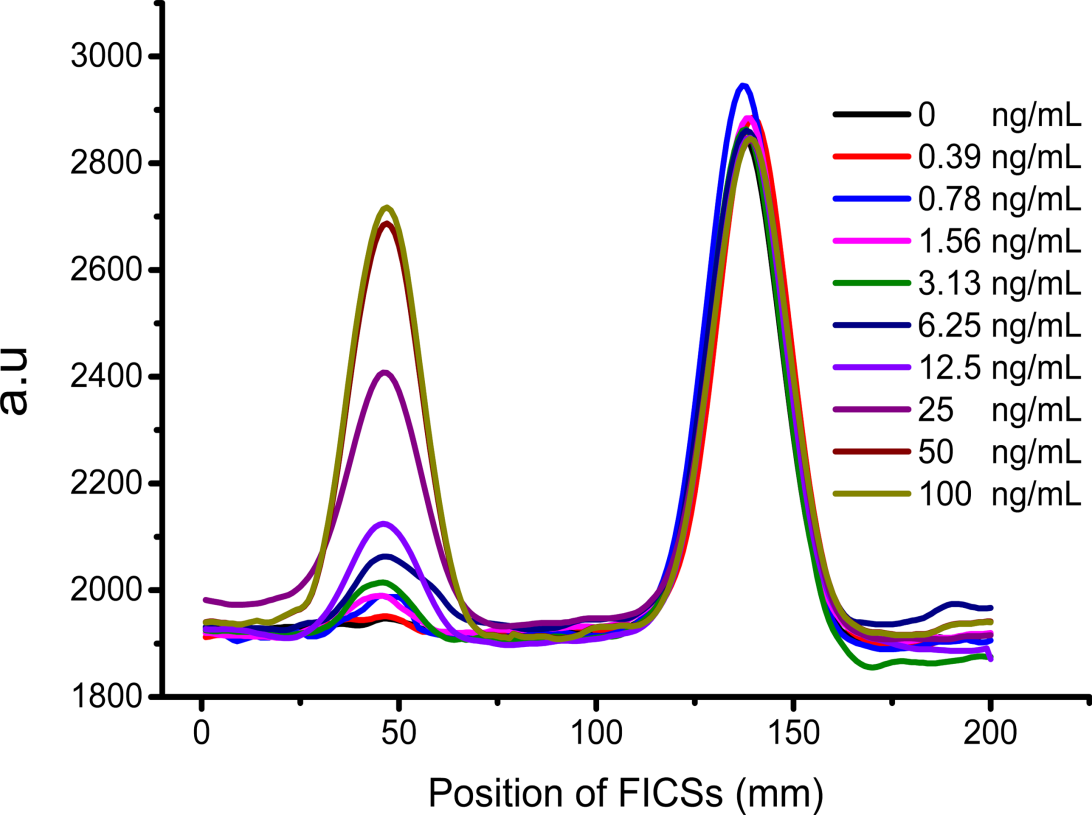
Figure S3.** Concentration-dependant fluorescence intensities of ICSs for CRP test.





**Figure S4.** Calibration curve of fluorescence ICSs for CRP test.


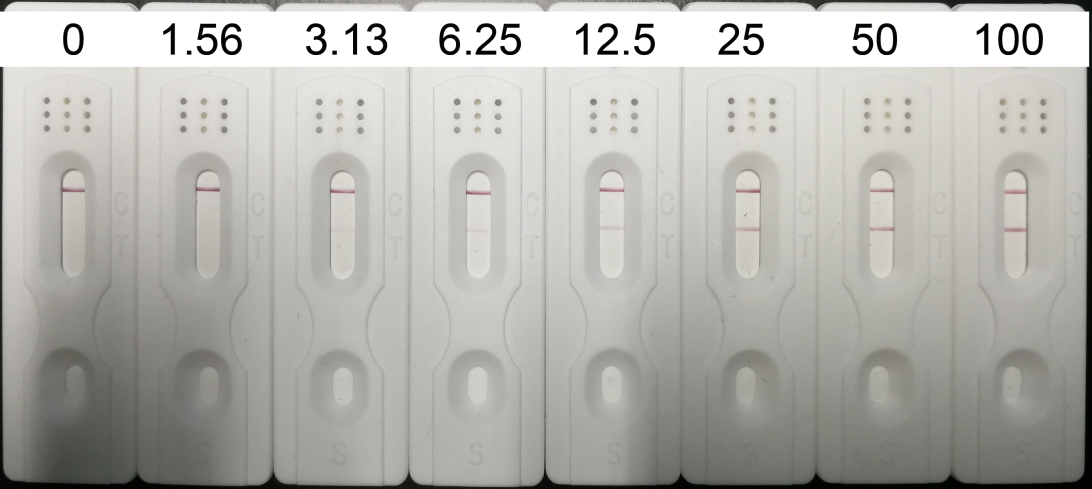


**Figure S5.** Results of AuNPs ICSs for CRP test.


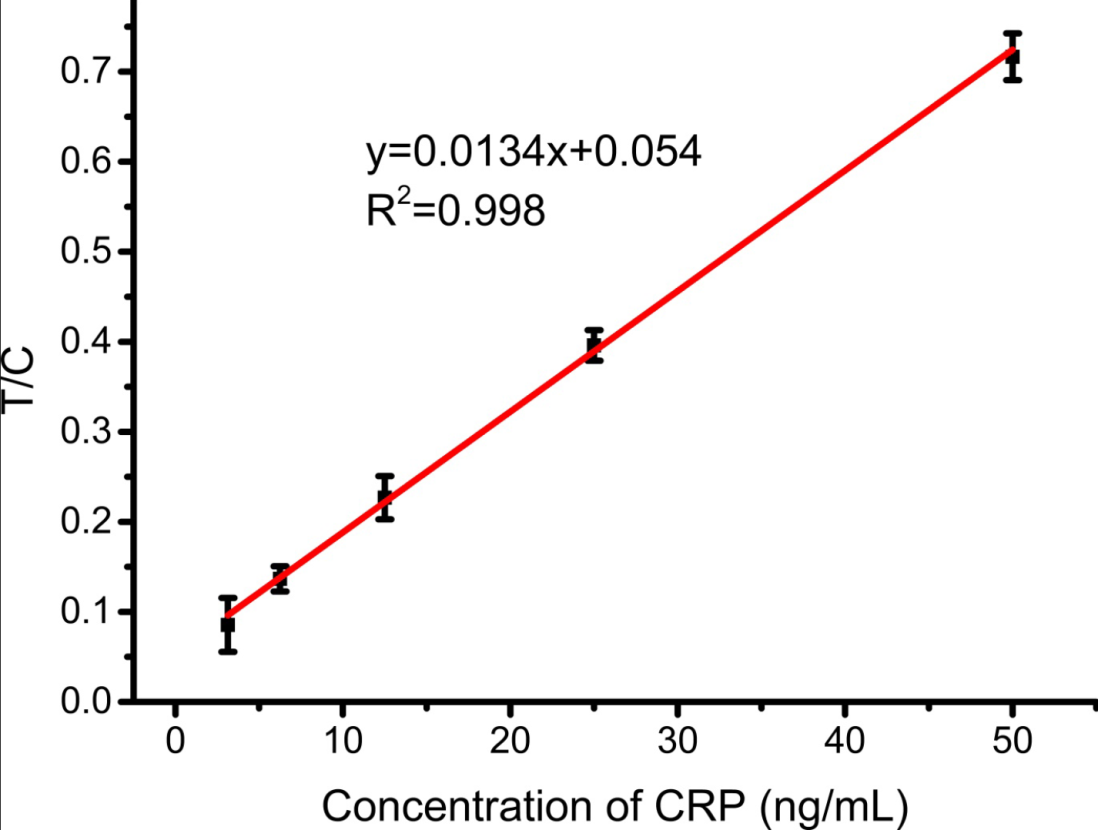


**Figure S6.** Calibration curve of AuNPs ICSs for CRP test.



**Figure S7**. Storage time of G-ICSs for CRP test.

**Table S1.** **Comparison of CRP test in serum samples by G-ICSs and chemiluminescent immunoassay (CL).**

| Sample number | | 1 | 2 | 3 | 4 | 5 | 6 | 7 | 8 | 9 | 10 | 11 | 12 | 13 | 14 |
| --- | --- | --- | --- | --- | --- | --- | --- | --- | --- | --- | --- | --- | --- | --- | --- |
| CL | | 84.57 | 25.43 | 6.76 | 1.21 | 59.99 | 3.43 | 64.17 | 10.25 | 11.64 | 1.78 | 3.14 | 15.83 | 3.43 | 1.91 |
| C-ICSs | 1 | 94.1 | 22.0 | 6.89 | 1.07 | 59.1 | 3.43 | 66.4 | 11.6 | 11.6 | 1.71 | 3.05 | 17.4 | 3.64 | 1.92 |
|  | 2 | 74.6 | 26.2 | 7.73 | 1.20 | 52.6 | 3.43 | 62.6 | 11.0 | 11.6 | 1.81 | 3.64 | 14.7 | 3.64 | 1.92 |
|  | 3 | 70.3 | 23.3 | 7.30 | 1.20 | 66.4 | 3.64 | 79.0 | 11.6 | 12.3 | 1.81 | 3.05 | 14.7 | 3.85 | 1.81 |
|  | Mean (mg/L) | 80.8 | 23.80 | 7.50 | 1.16 | 58.10 | 3.50 | 69.33 | 11.40 | 11.83 | 1.78 | 3.24 | 15.60 | 3.71 | 1.88 |
|  | S.D. (mg/L) | 11.53 | 2.15 | 0.45 | 0.08 | 5.07 | 0.12 | 8.58 | 0.35 | 0.40 | 0.06 | 0.34 | 1.56 | 0.12 | 0.06 |
|  | CV (100%) | 14.27 | 9.02 | 6.07 | 6.49 | 8.73 | 3.46 | 12.38 | 3.04 | 3.41 | 3.25 | 10.49 | 9.99 | 3.27 | 3.37 |
|  | Recovery (100%) | 95.5 | 93.72 | 110.99 | 95.59 | 96.84 | 102.04 | 108.05 | 108.37 | 101.66 | 99.81 | 103.40 | 98.55 | 103.34 | 98.60 |

**Note.** Mean: Average of CRP concentrations in serum samples test by the G-ICSs (n = 3). S.D.: Standard deviation of CRP concentration in serum samples test by the G-ICSs (n = 3). Coefficient of variation (CV) = (S.D./Mean)×100%. Recovery (%) = (Results of G-ICSs/Results of CL)*100%.

**

Figure S8.** Specificity of G-ICSs for CRP testing.

**

Figure S9.** Non-specific binding occurs when using G-ICSs to detect CRP at serum.
